# Clinical Significance of Keap1 and Nrf2 in Oral Squamous Cell Carcinoma

**DOI:** 10.1371/journal.pone.0083479

**Published:** 2013-12-27

**Authors:** Cong-Fa Huang, Lu Zhang, Si-Rui Ma, Zhi-Li Zhao, Wei-Ming Wang, Ke-Fei He, Yi-Fang Zhao, Wen-Feng Zhang, Bing Liu, Zhi-Jun Sun

**Affiliations:** 1 The State Key Laboratory Breeding Base of Basic Science of Stomatology & Key Laboratory of Oral Biomedicine Ministry of Education, School and Hospital of Stomatology, Wuhan University, Wuhan, China; 2 Department of Oral Maxillofacial-Head Neck Oncology, School and Hospital of Stomatology, Wuhan University, Wuhan, China; UAE University, Faculty of Medicine & Health Sciences, United Arab Emirates

## Abstract

Oxidative stress has been reported to play an important role in progression and prognostication in various kinds of cancers. However, the role and clinical significance of oxidative stress markers Keap1 and Nrf2 in oral squamous cell carcinoma (OSCC) has not been elucidated. This study aimed to investigate the correlation of oxidative stress markers Keap1 and Nrf2 expression and pathological features in OSCC by using tissue microarray. Tissue microarrays containing 17 normal oral mucosa, 7 oral epithelial dysplasia and 43 OSCC specimens were studied by immunohistochemistry. The association among these proteins and pathological features were analyzed. Expression of oxidative stress markers Keap1, Nrf2, and antioxidants PPIA, Prdx6, as well as CD147 was found to increase consecutively from normal oral mucosa to OSCC, and the Keap1, Nrf2, PPIA, Prdx6, CD147 expression in OSCC were significantly higher when compared to normal oral mucosa. Expression of Keap1, Nrf2 in tumors was not found to be significantly associated with T category, lymph node metastases, and pathological grade. Furthermore, we checked the relationship among these oxidative stress markers and found that Keap1 was significantly correlated with Nrf2, Prdx6 and CD147. Significant relationship between Nrf2 and Prdx6 was also detected. Finally, we found patients with overexpression of Keap1 and Nrf2 had not significantly worse overall survival by Kaplan–Meier analysis. These findings suggest that ROS markers are associated with carcinogenesis and progression of OSCC, which may have prognostic value and could be regarded as potential therapeutic targets in OSCC.

## Introduction

Head and neck squamous cell carcinoma (HNSCC) ranks sixth among the most common cancers in the world, it has a high propensity to metastasize to locoregional lymph nodes due to the presence of a rich lymphatic network and the overall high number of lymph nodes in the neck region [1]. Despite intensive research, the prognosis of HNSCC patients remains poor, with a 50% 5-year overall survival (OS) rate remains relatively unchanged for the past 3 decades [2]. Oral squamous cell carcinoma (OSCC) is the most frequent type of cancer of the head and neck area. Better understanding of the molecular mechanisms regulating tumor invasion and metastasis for OSCC may lead to more effective treatment options.

One of the crucial cancer characteristics that contribute to the worse survival of OSCC is altered intracellular environment. Oxidative stress, implicated in the etiology of cancer, occurs when there is a significant imbalance between the production and removal of reactive oxygen species (ROS), resulting in irreversible oxidative damage to DNA and proteins, interfering with important cellular function [3]. In recent years, it is widely recognized that increased ROS lead to signaling pathways activation that benefit cancer cells for initiation, survival and progression [4]. Nrf2 (Nuclear Factor-E2-related factor 2), as a transcription factor, functions as regulate the expression of a plenty of antioxidant proteins. Kelch-like ECH-associated protein-1 (Keap1) is an oxidative stress sensor, mediating degradation of Nrf2, the later is a well known substrate for Keap1 [5]. In the absence of Keap1, Nrf2 is constitutively stabilized, and the expression of Nrf2 target genes is maintained at high levels [5]. Overexpressed Nrf2 activates the antioxidant response and induce transcription of a wide array of genes, which are able to combat the harmful effects of oxidative stress. Keap1/Nrf2 signaling pathway of have been identified to have important roles in the cellular response to oxidative stress, electrophiles and xenobiotics [6], [7]. KEAP1 mutations lead to constitutively active Nrf2 and subsequent protection of cancer cells from chemotherapeutic drugs, constitutive activation of Nrf2 is prominently expressed in various kinds of cancers, which has been shown to protect against cancer and leads to progression and poor survival [6], [7].

Although little is known about the function of antioxidant enzymes in cancer cells, it has been recently reported that antioxidant enzymes including glutathione S-transferase, glutathione peroxidase, and NADH quinine oxidoreductase-1, peroxiredoxins, thioredoxin, PPIA (cyclophilin A) protects cancer cells against oxidative stress induced apoptosis, as well as hypoxia and chemotherapy [8]. We have investigated the expression of peroxiredoxin6, PPIA, thioredoxin and thioredoxin reductase-1 in tongue squamous cell carcinoma, and found that overexpression of PPIA and thioredoxin 1 were correlated to worse survival in our previous studies [9]–[11]. CD147, a widely distributed cell surface glycoprotein, is identified as a signalling receptor to extarcellular PPIA. Both of PPIA and CD147 are highly expressed in cancer cells and associated to promoting cancer invasiveness and chemoresistance [12], [13]. However, the specific mechanism between oxidative stress, antioxidant enzymes, CD147 and OSCC is unclear, and the roles of these proteins in OSCC have not been clarified.

To date, no comprehensive analysis has been performed of Keap1 and Nrf2 expression and associated genetic abnormalities in OSCC, and no studies have determined the association between the expression of Keap1 and Nrf2 and clinical outcome. In this study, our aim was to examine the expression of Nrf2 pathway in OSCC by employing tissue microarray, and analyze the association among these factors. Finally, the relevance of the expression levels of these proteins with pathologic features and clinical outcome are further evaluated with an aim to decide their roles in the prognosis of OSCC.

## Materials and Methods

### Ethics Statement

This study was approved by the Medical Ethics Committee of Hospital of Stomatology Wuhan University (PI: Zhi-Jun Sun), and informed written consent to participate in the study was obtained from each individual before surgery.

### Patient Samples

To determine the expression of Keap1 and Nrf2 in OSCC, we selected archived, formalin-fixed, paraffin-embedded (FFPE) tumor tissue samples from primary surgically resected oral cancer specimens from the department of Oral and Maxillofacial Surgery, School and Hospital of Stomatology Wuhan University (Table 1). The procedures were performed in accordance with the National Institutes of Health guidelines regarding the use of human tissues. Tumor tissues were histologically analyzed and classified using the World Health Organization classification system, the clinical stages of the OSCC were classified according to the guidelines of the International Union against Cancer (UICC 2002). Recurrence OSCC, primary OSCC with induced chemotherapy and/or radiotherapy are excluded in this study. The medium follow-up period was 24 months (range from 12–43 months).

**Table 1:** Cinicopathological features of 43 oral squamous cell carcinoma used in this study.

*Core*	*Location*	*Tumor(cm)*	*TNM*	*Grade*
A1	Tongue	3.0*2.0	T2N0M0	I
A2	Gingiva	2.0*1.5	T1N1M0	I
A3	Tongue	1.5*1.5	T1N1M0	I
A4	Tongue	2.0*2.0	T2N0M0	I
A5	Gingiva	3.0*2.0	T2N0M0	I
A6	Tongue	1.7*1.7	T1N0M0	I
A7	Tongue	3.0*1.5	T2N1M0	I
A8	Tongue	2.0*2.0	T1N0M0	I
A9	Gingiva	3.0*2.0	T2N0M0	I
B1	Buccal mucosa	3.0*2.0	T2N1M0	I
B2	Buccal mucosa	5.0*5.0	T3N0M0	I
B3	Tongue	2.0*2.0	T1N0M0	I
B4	Buccal mucosa	3.0*3.0	T2N0M0	II
B5	Buccal mucosa	4.0*3.0	T3N1M0	II
B6	Buccal mucosa	5.0*4.0	T3N0M0	II
B7	Tongue	3.0*2.0	T2N1M0	II
B8	Tongue	1.5*1.5	T1N0M0	II
B9	Tongue	4.0*3.0	T3N0M0	II
B10	Tongue	5.0*4.0	T3N0M0	II
C1	Tongue	3.0*2.0	T2N0M0	II
C2	Tongue	3.0*2.0	T2N1M0	II
C3	Tongue	1.5*1.0	T1N1M0	II
C4	Tongue	3.0*2.0	T2N1M0	II
C5	Tongue	3.0*2.0	T2N1M0	II
C6	Tongue	3.0*2.5	T2N0M0	II
C7	Tongue	4.0*2.0	T3N0M0	II
C8	Buccal mucosa	4.0*3.0	T2N1M0	II
C9	Tongue	2.0*2.0	T2N1M0	II
C10	Tongue	3.0*2.0	T2N1M0	II
D1	Oropharyngeal	3.0*3.0	T2N0M0	II
D2	Mouth floor	2.0*2.0	T2N0M0	II
D3	Gingiva	3.0*1.5	T2N0M0	III
D4	Buccal mucosa	4.0*3.0	T3N1M0	III
D5	Tongue	2.0*1.0	T2N0M0	III
D6	Buccal mucosa	3.0*2.5	T2N1M0	III
D7	Gingiva	3.0*2.5	T2N0M0	III
D8	Gingiva	3.0*2.0	T2N0M0	III
D9	Tongue	5.0*3.0	T3N1M0	III
D10	Mouth floor	3.0*3.0	T2N1M0	III
E1	Tongue	5.0*3.0	T3N1M0	III
E2	Tongue	5.0*4.0	T3N0M0	III
E3	Mouth floor	4.0*4.0	T3N0M0	III
E4	Gingiva	3.0*3.0	T2N1M0	III

### Tissue Microarray

Custom made tissue microarrays (T12–412) were constructed using the block mentioned above including 43 OSCC, 17 normal oral mucosa and 7 oral epithelial dysplasia were selected as control group. The cases were selected based on the availability of FFPE tissue blocks with enough tumor tissue for TMA construction. Detailed clinicopathologic information, including smoking history, T category, lymph node metastasis, TNM stage, histologic grade were available for all cases.

### Immunohistochemistry (IHC)

Immunohistochemical studies of the human OSCC tissue microarrays were done using the following antibodies: polyclonal rabbit anti-human Nrf2 (1∶200) from Epitomics Biotechnology, Inc., (Burlingame, USA); monoclonal mouse anti-human Prdx6 (dilution 1∶1000) from Abcam Biotechnology Inc., (Cambridge, UK), monoclonal mouse anti-human Keap1 and polyclonal rabbit anti-human PPIA, CD147 (dilution 1∶400, 1∶100 and 1∶200, respectivety) from Proteintech Group Inc., (Chicago, USA).

Tissue sections were deparaffinized and hydrated, and antigen retrieval was performed in pH 6.0 citrate buffer. After cooling down to room temperature, sections were incubated in 3% hydrogen peroxide to quench the endogenous peroxidase activity. Then sections were incubated at 4°C overnight with primary antibody and washed with Tris buffer, followed by incubation with biotin-labeled secondary antibody for 15 minutes and streptavidin peroxidase for 15 minutes. After sections adequate elusion with PBS, Diaminobenzidine as well as a counterstaining with haematoxylin resulted in the visualization of the immunostaining.

### Scoring System

Slices were scanned using an Aperio ScanScope CS scanner (Vista, CA, USA) with background substrate for each slice, and quantified using Aperio Quantification software (Version 9.1) for membrane, nuclear, or pixel quantification as previous described [14]. An area of interest was selected either in the epithelial or the cancerous area for scanning and quantification. Histoscore of membrane and nuclear staining was calculated as a percentage of different positive cells using the formula (3+)×3+(2+)×2+(1+)×1 [14]. Histoscore of pixel quantification was calculated as total intensity/total cell number. The threshold for scanning of different positive cells was set according to the standard controls provided by Aperio.

### Hierarchical Clustering, Data Visualization, and Statistical Analysis

In Microsoft excel, the staining scores were converted into scaled values centered on zero, then the Cluster 3.0 with average linkage based on Pearson’s correlation coefficient was used to achieve the hierarchical analysis [15], and the results were visualized using the Java TreeView 1.0.5 [16]. Finally, the clustered data were arranged with markers on the horizontal axis and tissue samples on the vertical axis. Biomarkers with a close relationship are located next to each other.

### Statistical Analysis

Statistical data analysis was performed with GraphPad Prism 5.03 (GraphPad Software, Inc., La Jolla, CA) statistical packages. The differences in immunostaining and protein levels among each group were analyzed by the One-way ANOVA followed by the post-Turkey or Bonferroni multiple comparison tests. Two-tailed Pearson statistics were used for correlated expression of these markers after confirmation of the sample with Gaussian distribution. Survival curves were plotted using the method of Kaplan-Meier and the significance of observed differences was assessed with log-rank test. Statistical significance was defined as the p-value was <0.05.

## Results

### Expression of Keap1, Nrf2, PPIA, Prdx6 and CD147 in Normal Oral Mucosa, Oral Epithelial Dysplasia and OSCC

**Figure 1:**
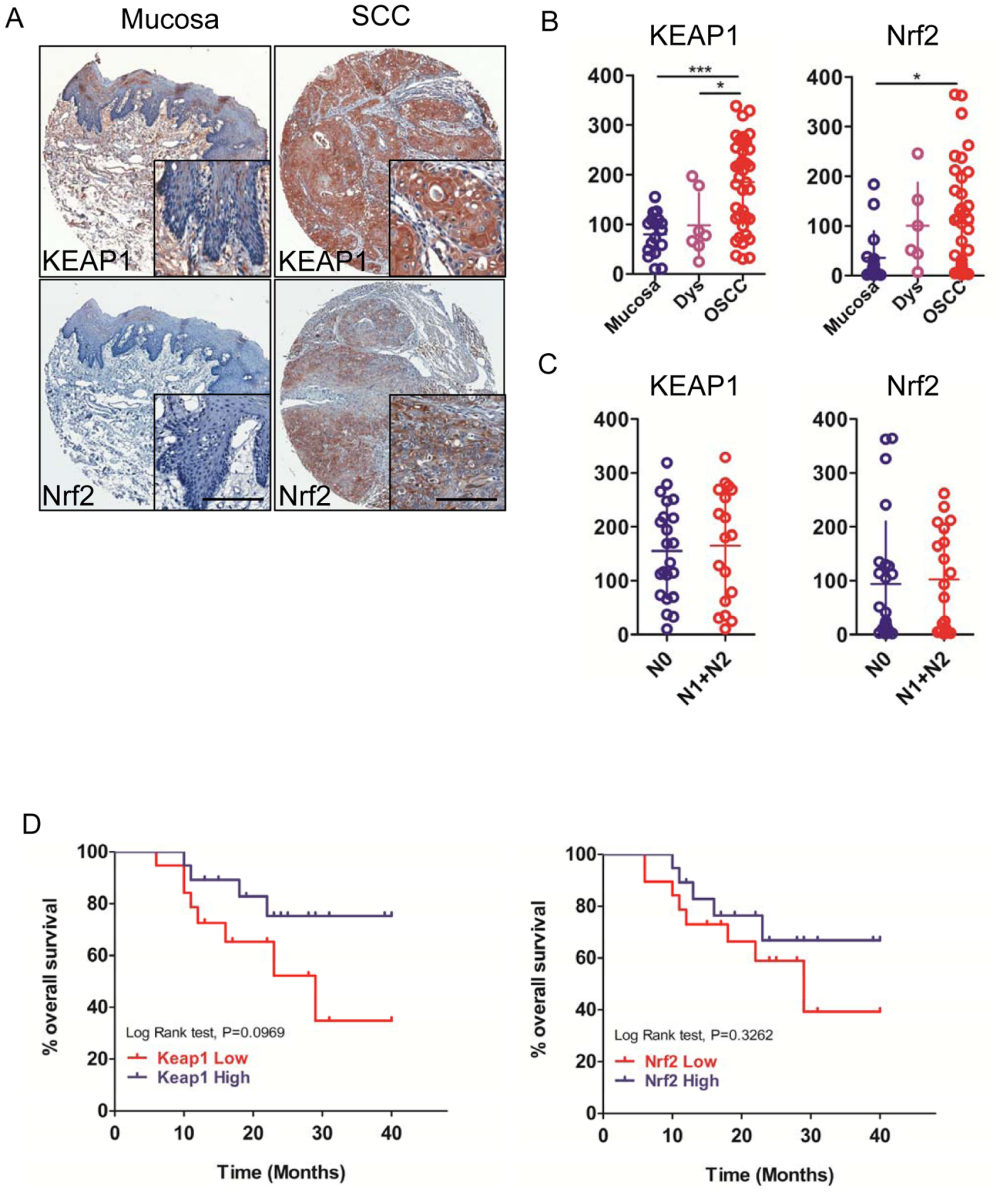
Keap1 and Nrf2 were overexpressed in human OSCCs. A, Representative immunohistochemical staining of Keap1 and Nrf2 in human oral cancer tissue (right) compared with normal oral mucosa (left) (Scale bars = 100 umol). B, Quantatitive of histoscore of Keap1 and Nrf2 expression in normal oral mucosa, oral epithelial dysplasia and human oral cancer, Keap1 levels in OSCC was significant higher when compared with oral epithelial dysplasia or normal oral mucosa, and Nrf2 levels in OSCC was significant higher when compared with normal oral mucosa (Mean ± SEM; *, *P*<0.05; **, *P*<0.01; ***, P<0.001; One-way ANOVA). C, The expression of Keap1 and Nrf2 were not correlated with lymph node status of human oral cancer (Quantification using Aperio nuclear quantification software, and statistics using Graph Pad Prism 5. Mean ± SEM; *, *P*<0.05; Mann–Whitney U test). D, Overall survival of the OSCC patients with Keap1 and Nrf2 expression calculated and presented by Kaplan–Meier analysis, and both of Keap1 and Nrf2 expression were not significantly correlated with overall survival (*P>0.05*).

**Figure 2:**
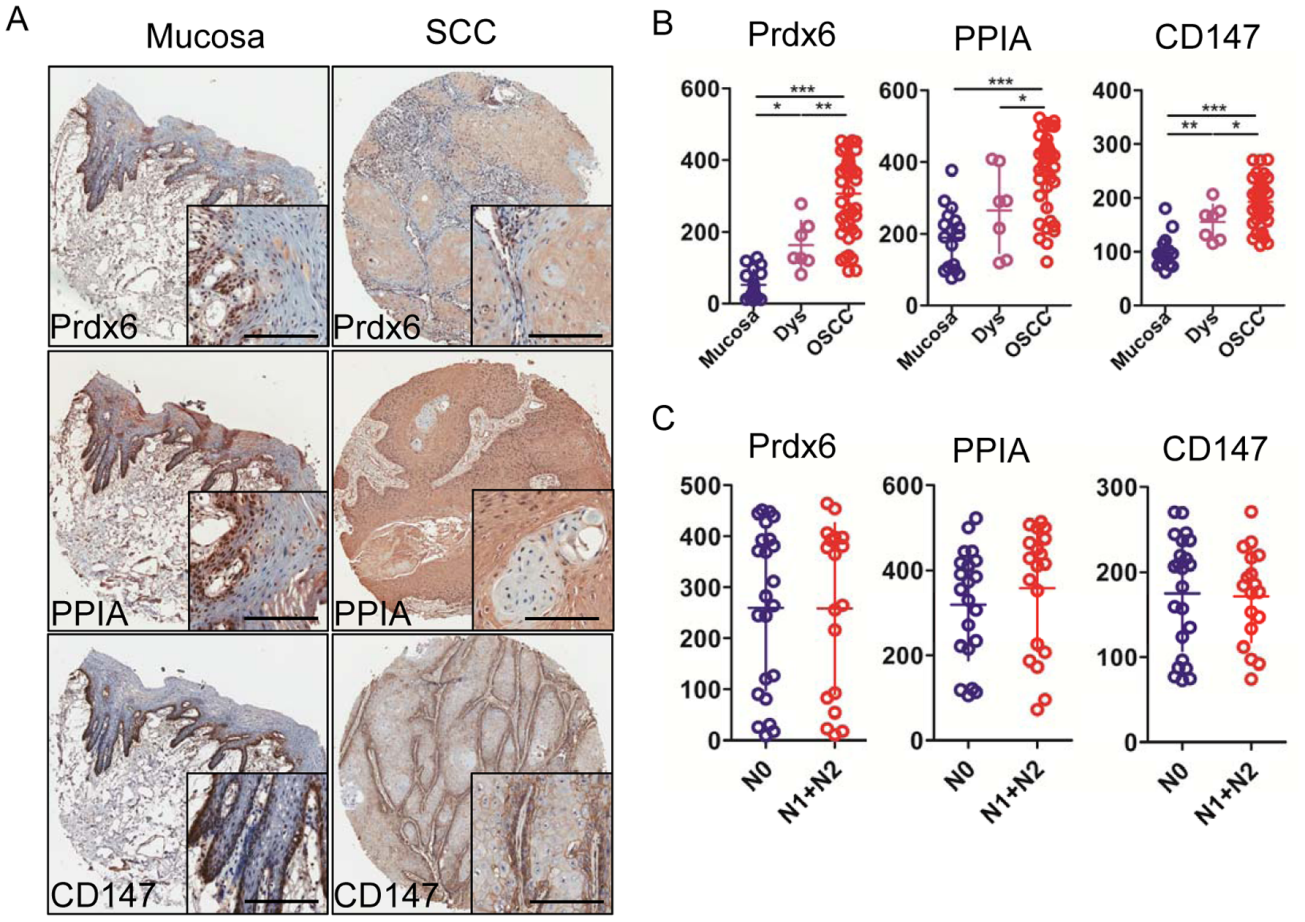
Prdx6, PPIA and CD147 were overexpressed in human OSCCs. A, Representative immunohistochemical staining of Prdx6, PPIA and CD147 in human oral cancer tissue (right) compared with normal oral mucosa (left) (Scale bars = 100 umol). B, Quantatitive of histoscore of Prdx6, PPIA and CD147 expression in normal oral mucosa, oral epithelial dysplasia and human oral cancer, Prdx6, PPIA and CD147 levels in OSCC was significant higher when compared with oral epithelial dysplasia and/or normal oral mucosa (Mean ± SEM; *, *P*<0.05; **, *P*<0.01; ***, *P*<0.001; One-way ANOVA). C, Prdx6, PPIA and CD147 were not significantly associated with lymph node status (Quantification using Aperio nuclear quantification software, and statistics using Graph Pad Prism 5. Mean ± SEM; *, *P*<0.05; Mann–Whitney U test and One-way ANOVA).

To assess the expression of the oxidative stress markers Keap1, Nrf2, and antioxidants PPIA, Prdx6, as well as CD147 in human OSCCs, we stained the tumor sections from human tissue arrays for OSCC (n = 43) with antibodies for Keap1, Nrf2, PPIA, Prdx6 and CD147 compared them with oral epithelial dysplasia (n = 67) and normal oral mucosa samples (n = 17). For the study of the expression of these markers in OSCC TMAs and whole section tissue specimens, we focused on nuclear Nrf2 expression because this is the subcellular location where is considered to be biologically active [17], [18], and on cytoplasmic Keap1 expression because it was the only expression detected in malignant tumor cells in the tissue specimens. We found that Nrf2 nuclear expression was significantly increased when compare with the normal oral epithelium (*P*<0.05) (Fig. 1). Then the expression of keap1 was mainly founded significantly increased in cytoplasm, as compared with oral epithelial dysplasia or normal oral mucosa (*P*<0.05). The expression of PPIA, Prdx6, and CD147 have been studied in our previous researches in OSCC, and this is the first time to assess their expression with using the automated analysis on a microarray. The data also showed us that the level of PPIA, Prdx6, and CD147 in OSCC was significant higher when compared with oral epithelial dysplasia and/or normal oral mucosa (*P*<0.05) (Fig. 2).

### Correlation between these Marker Expression, Pathologic Features, and Stage of Disease

In order to authenticate the relationship between antioxidants PPIA and Prdx6, and oxidative stress markers Nrf2, Keap1, as well as CD147 with pathologic features. One-way ANOVA and the Mann–Whitney U test were used, Table 2 showed the association of several clinicopathologic factors with Keap1, Nrf2, PPIA, Prdx6 and CD147. Overall, The data show us that Keap1, Nrf2, PPIA, Prdx6 and CD147 were not correlated with tumor stage (T1 to T3), lymph node status (N0 to N1), pathological grade (GI to GIII) (*P*>0.05) (Fig. S1, Fig. S2).

**Table 2:** Pearson correlation coefficient test analyses of the array immunostainings of Keap1, Nrf2, PPIA, Prdx6, CD147 in OSCC (n = 43).

*Markers*	*Nrf2*	*PPIA*	*Prdx6*	*CD147*
Keap1	*P* = 0.000, R = 0.549	*P* = 0.264, R = 0.148	*P* = 0.000, R = 0.735	*P* = 0.040, R = 0.268
Nrf2		*P* = 0.109, R = 0.211	*P* = 0.000, R = 0.551	*P* = 0.353, R = 0.123

### The Relationships among Expression of Keap1, Nrf2, PPIA, Prdx6 and CD147

**Figure 3:**
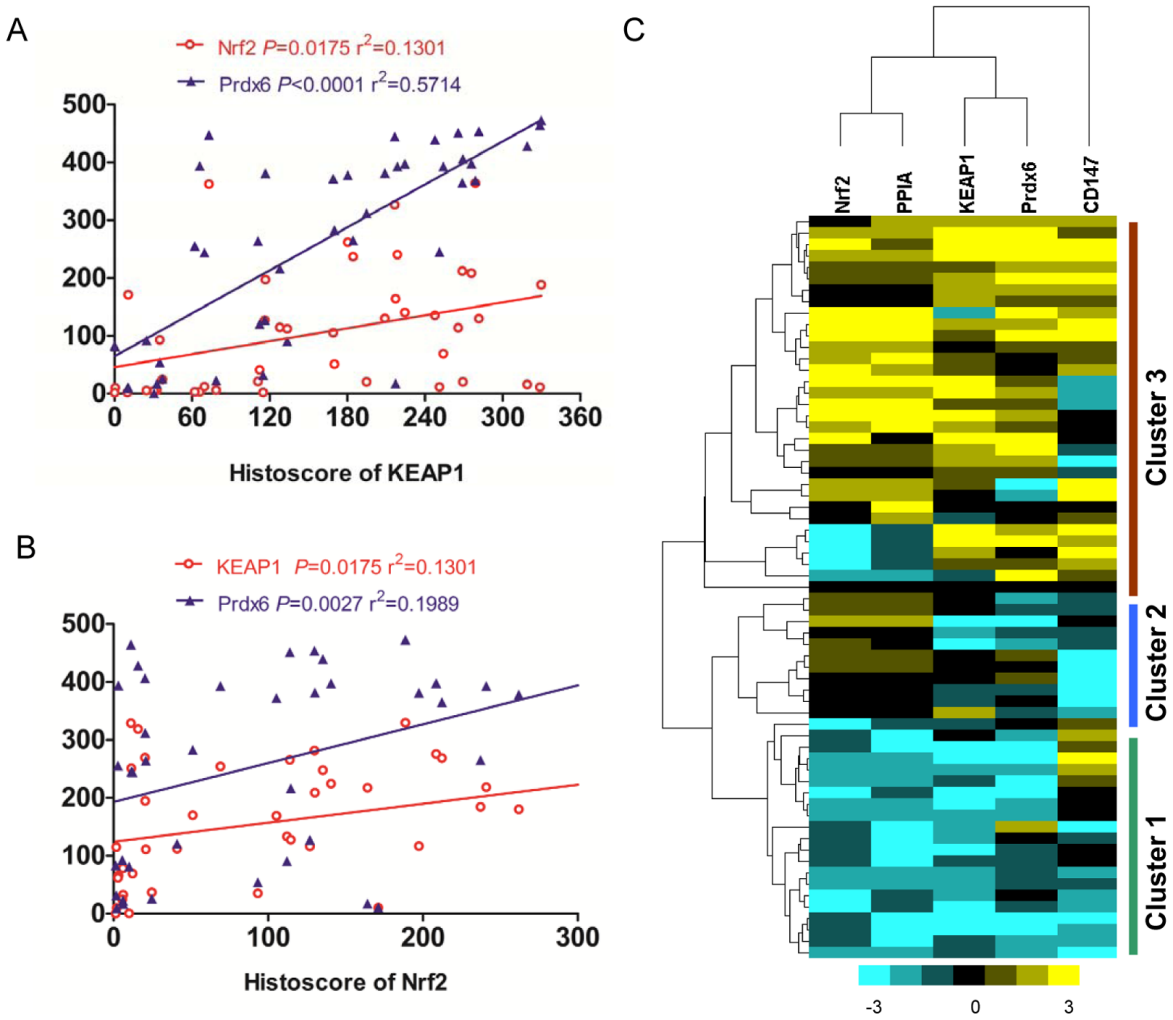
Correlation of Keap1, Nrf2 with PPIA, Prdx6 and CD147 in human OSCC tissue array. A, The expression of Keap1 had significant correlations with Nrf2 (P = 0.000, R = 0.549), and Prdx6 (P = 0.000, R = 0.735), and CD147 (P = 0.040, R = 0.268) by using the Pearson correlation coefficient test in human OSCC tissue array. B, The expression of Nrf2 was closely correlated with Keap1 (P = 0.000, R = 0.549) and Prdx6 (P = 0.000, R = 0.551) in human OSCC tissue array. C, Hierarchical Clustering of Keap1, Nrf2 with PPIA, Prdx6 and CD147 in human OSCC tissue array. Histoscore based on quantification using Aperio quantification software and statistics with Graph Pad Prism5. Mean ± SEM; 2-tailed Pearson correlation statistics.

To determine the biological effect of Keap1/Nrf2 pathway in OSCC malignant cells, we studied the correlation of expression of nuclear Nrf2 with the immunohistochemical protein expression of antioxidants PPIA and Prdx6, as well as CD147, the Spearman rank correlation coefficient test and linear tendency test was used. Of interest, we found that the expression of higher cytoplasmic Keap1 was statistically associated with nuclear Nrf2 (*P* = 0.000, *R* = 0.549), and cytoplasmic Prdx6 (*P* = 0.000, R = 0.735), and membrane CD147 (*P = *0.040, R = 0.268), However, the relationship between cytoplasmic Keap1 was not found significantly with PPIA (*P* = 0.264, R = 0.148). Surprisingly, Nrf2 was closely correlated with Prdx6 (*P* = 0.000, R = 0.551), and no relationship was found between the expression of PPIA (*P* = 0.109, R = 0.211) and CD147 (*P* = 0.353, R = 0.123) (table 2). By hierarchical clustering, the expression of tumor associated antioxidants PPIA and Prdx6, and oxidative stress marker Nrf2 is more close to expression of Keap1 (Fig. 3).

### Association between Keap1 and Nrf2 Expression and OSCC Patient Outcome Using TMA Specimens

We determined the association between cytoplasmic Keap1 and nuclear Nrf2 expression and over all survival rates in patients with OSCC. Kaplan–Meier method was employed to analysis the relationship between overall survival and Keap1 and Nrf2. Follow-up information was available on 43 patients with OSCC ranging from 12 months to 43 months. At the end of this study, 6 patiens were lost during follow up, 29 patients were alive, 10 patients suffered from a recurrence, 8 had died of cancer, the 2-year overall and disease-free survival rate were 78.4% and 70.3%, respectively. Figure 2 showed the survival curves stratified according to Keap1 and Nrf2 expression. Nevertheless, cumulative rate of the patients with cytoplasmic Keap1 expression were not significantly correlated with overall survival (*P*>0.05). Similarly, no association was found between nuclear Nrf2 expression and outcome in patients with OSCC (*P*>0.05).

## Discussion

Oxidative stress is caused by an imbalance of ROS and the antioxidative stress defense systems, which is well recognized as one of the chief risk factors for carcinogenesis. Recently, oxidative stress is reported to correlate with cancer progression and the response to therapy [19]. Despite all these recent findings, the specific mechanisms between oxidative stress, redox homeostasis and the activation of carcinogenesis pathways in oral cancer are not well understood.

The Keap1-Nrf2 system plays a crucial role in cellular defense against oxidative stress, which has been reported to promote cancer development and resistance to chemotherapeutic drugs [20], but little is known about its association with carcinogenesis of oral cancer. We performed a comprehensive immunohistochemical analysis of Keap1 and Nrf2 expression in OSCC tumors. In the present study, our results showed that Nrf2 was mainly located in nuclear, and its expression score increased progressively from normal oral mucosa to oral epithelial dysplasia and OSCC, but it was different between OSCC and oral mucosa. Nuclear Nrf2 expression has been reported at different frequencies in other epithelial tumors, including squamous cell carcinoma of the head and neck [17], [18] and lung cancer. [21]. Keap1, as a binding partner, is an adaptor of the ubiquitin ligase complex, which is essential for the regulation of activity of Nrf2. In pancreatic cancer and colorectal cancer [22], [23], Keap1 expression was significantly increased, our results was accordance with their researches. Recently, evidences showed a high incidence occurrence of loss of Keap1 function in cancer, Keap1 mutations affected the repressive activity of Keap1 against Nrf2, the loss of Keap1 function enhanced the nuclear accumulation of Nrf2 and elevated the expression of antioxidative and antixenobiotic stress enzymes and drug efflux pumps [24]. In fact, the Keap1–Nrf2 pathway plays in the protection of our body against drug toxicity and stress induced diseases, and cancer cells hijack Nrf2 activity to support their malignant growth and thus Nrf2 has emerged as a therapeutic target.

Furthermore, as the ROS lever was changed all the time in the cancer progression, then the lever of antioxidant enzymes was also changed followed the ROS level, and Nrf2 neutralizes ROS to restore cellular redox balance [17], [18]. To investigate the relationships between Keap1-Nrf2 system and antioxidant enzymes, we used the Spearman rank correlation coefficient test and linear tendency test and found that Keap1 had significant correlations with Nrf2, Prdx6 and CD147 except PPIA. In addition, Nrf2 was closely correlated with Keap1 and Prdx6, but not PPIA and CD147. PPIA and Prdx6 were antioxidant enzymes, and CD147 was the receptor of PPIA, all of them had been researched in our previous studies [9]–[11], and this study showed that the expression scores of PPIA, Prdx6 and CD147 increased progressively from normal oral mucosa to OSCC, which indicated that the antioxidant enzymes was regulated following the cancer development. Nrf2 is one of the key transcription factors for Prdx1 gene expression, increased nuclear localization and transactivation of Nrf2 by hypoxia/reoxygenation was accompanied by a reduced level of Keap1 protein [25]. The association found between the expressions of nuclear Nrf2 with cytoplasmic Prdx6 protein in a subset of our OSCC, suggest that Nrf2 is biologically active in the nucleus of the OSCC cells and cytoplasmic Prdx6 might be regulated by nuclear Nrf2. To the best of our knowledge, this association has not been previously reported, and it warrants further study.

Ours is the first reported study to determine the frequency of low or high cytoplasmic Keap1 and nuclear Nrf2 expression in OSCC and their association with tumors’ clinicopathologic characteristics. Our findings differ from those of previous publications that Keap1 and nuclear Nrf2 were associated with more advanced tumor stage [26]. We found that Keap1, Nrf2, PPIA, Prdx6 and CD147 were not significantly correlated with tumor stage, lymph node status, pathological grade, but the results were same as in pancreatic cancer [22], [23], the association between the levels of Nrf2 or Keap1 with clinicopathological parameters was also not significant, which indicating that large number of cases may be needed to clarify in future study. We previously used immunohistochemistry methods to evaluate the prognostic role of members of antioxidant enzymes, overexpression of PPIA, CD147 and thioredoxin 1 were correlated to worse survival in tongue carcinoma [9]–[11]. Then in this study, we evaluated the immunohistochemical expression of nuclear Nrf2 and cytoplasmic Keap1 with outcome, but both of them were not associated with the outcome. According to other researches in non-small-cell lung cancer [27], Nrf2 and Keap1 were closely related to outcome and could be independent prognostic factors, we suggest that Nrf2 and Keap1 were correlated with oral cancer prognosis, and large number of cases study will be explored to identify this suggestion. In human lung cancer, tumors showing high levels of Nrf2 protein are associated with a poor outcome and increased resistance to therapy [24]. Thus, discovery and development of Nrf2 inhibitors based on the molecular mechanisms of the Keap1-Nrf2 system is one of the most critical and challenging assignments for conquering cancers. Since the sample size is limit in this study, a large scale of OSCC tissue may be needed to further confirm the diagnostic and prognostic role of KEAP1 and Nrf2 in OSCC progression.

In conclusion, abnormal expression of Keap1 and Nrf2 proteins was more common than that of the normal oral mucosa and oral epithelial dysplasia tissues, which was significantly related with Prdx6 and CD147, suggesting that Keap1-Nrf2 system and antioxidant enzymes could play an important role together on progression of human oral cancer and might serve as potential target molecules for cancer therapy. However, further studies are needed to clarify the mechanism by which Keap1-Nrf2 system is involved in the development and progression of oral cancer.

## Supporting Information

Figure S1 **The correlation between Keap1 and Nrf2 with pathologic features.** A, The correlation between the expression of Keap1 and Nrf2 with T category in OSCC. B, The correlation between the expression of Keap1 and Nrf2 with pathological grade in OSCC. Quantification using Aperio nuclear quantification software, and statistics using Graph Pad Prism 5. Mean±SEM; Mann–Whitney U test or One-way ANOVA.
(TIF)

Figure S2 **The correlation between PPIA, Prdx6 and CD147 with pathologic features.** A, The correlation between the expression of PPIA, Prdx6 and CD147 with T category in OSCC. B, The correlation between the expression of PPIA, Prdx6 and CD147 with pathological grade in OSCC. Quantification using Aperio nuclear quantification software, and statistics using Graph Pad Prism 5. Mean±SEM; Mann–Whitney U test or One-way ANOVA.
(TIF)
