# Supplementary figures and images for: Clinical Significance of Keap1 and Nrf2 in Oral Squamous Cell Carcinoma
Source: PLoS One. 2013 Dec 27;8(12):e83479. doi: 10.1371/journal.pone.0083479 (PMC3873935; doi:10.1371/journal.pone.0083479)

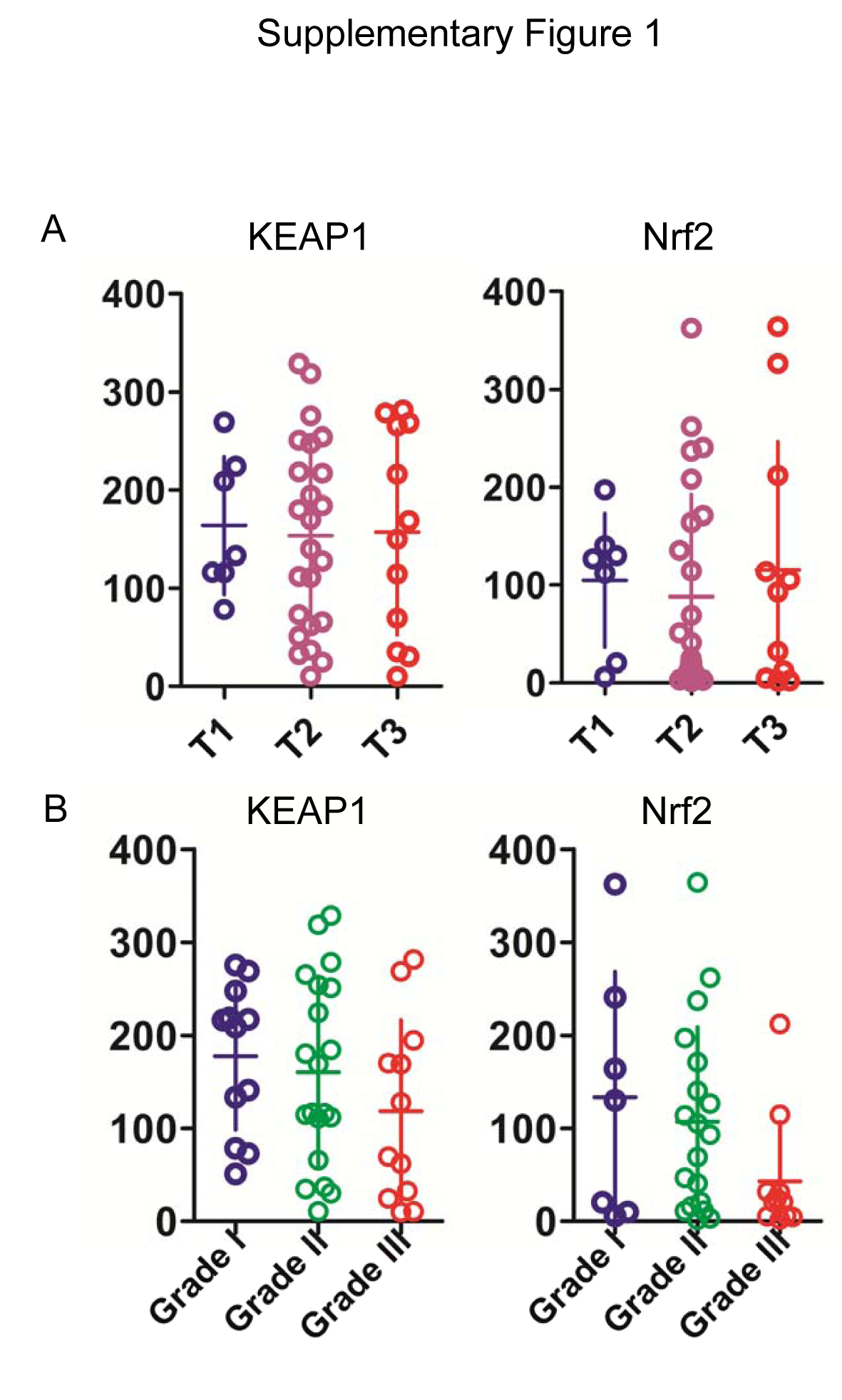

Supplement: Figure S1 — The correlation between Keap1 and Nrf2 with pathologic features. A, The correlation between the expression of Keap1 and Nrf2 with T category in OSCC. B, The correlation between the expression of Keap1 and Nrf2 with pathological grade in OSCC. Quantification using Aperio nuclear quantification software, and statistics using Graph Pad Prism 5. Mean±SEM; Mann–Whitney U test or One-way ANOVA. (TIF) [file pone.0083479.s001.tif]

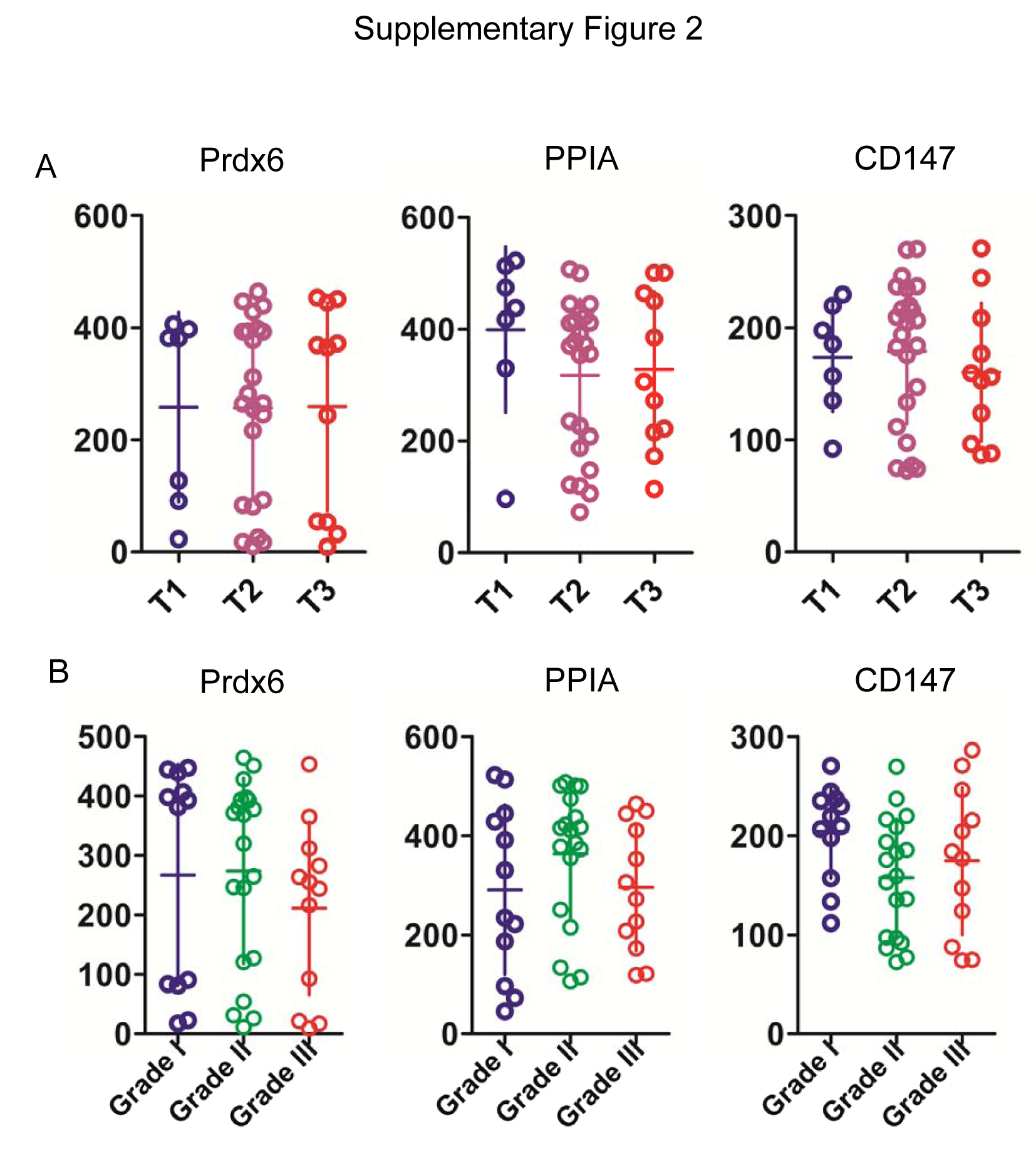

Supplement: Figure S2 — The correlation between PPIA, Prdx6 and CD147 with pathologic features. A, The correlation between the expression of PPIA, Prdx6 and CD147 with T category in OSCC. B, The correlation between the expression of PPIA, Prdx6 and CD147 with pathological grade in OSCC. Quantification using Aperio nuclear quantification software, and statistics using Graph Pad Prism 5. Mean±SEM; Mann–Whitney U test or One-way ANOVA. (TIF) [file pone.0083479.s002.tif]
